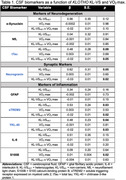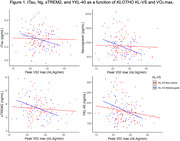# KLOTHO KL‐VS genotype modifies the relationship between cardiorespiratory fitness and CSF biomarkers of neurodegeneration, synaptic dysfunction, and inflammation

**DOI:** 10.1002/alz.086169

**Published:** 2025-01-09

**Authors:** Mackenzie Jarchow, Ira Driscoll, Brianne M. Breidenbach, Noah R. Cook, Catherine L. Gallagher, Sterling C. Johnson, Sanjay Asthana, Bruce P Hermann, Mark A. Sager, Kaj Blennow, Henrik Zetterberg, Cynthia M. Carlsson, Gwendlyn Kollmorgen, Clara Quijano‐Rubio, Dane B. Cook, Dena B. Dubal, Ozioma C Okonkwo

**Affiliations:** ^1^ Wisconsin Alzheimer's Disease Research Center, School of Medicine and Public Health, University of Wisconsin‐Madison, Madison, WI USA; ^2^ Wisconsin Alzheimer's Institute, Madison, WI USA; ^3^ School of Medicine and Public Health, University of Wisconsin‐Madison, Madison, WI USA; ^4^ Geriatric Research Education and Clinical Center William S. Middleton VA Hospital, Madison, WI USA; ^5^ Wisconsin Alzheimer's Disease Research Center, Madison, WI USA; ^6^ Wisconsin Alzheimer's Institute, University of Wisconsin School of Medicine and Public Health, Madison, WI USA; ^7^ Paris Brain Institute, ICM, Pitié‐Salpêtrière Hospital, Sorbonne University, Paris France; ^8^ Clinical Neurochemistry Laboratory, Sahlgrenska University Hospital, Mölndal Sweden; ^9^ Institute of Neuroscience and Physiology, Sahlgrenska Academy at the University of Gothenburg, Göteborg Sweden; ^10^ Neurodegenerative Disorder Research Center, Institute on Aging and Brain Disorders, University of Science and Technology of China and First Affiliated Hospital of USTC, Heifei China; ^11^ Hong Kong Center for Neurodegenerative Diseases, Hong Kong China; ^12^ Institute of Neuroscience and Physiology, Sahlgrenska Academy at the University of Gothenburg, Mölndal, Gothenburg Sweden; ^13^ UCL Institute of Neurology, Queen Square, London United Kingdom; ^14^ Roche Diagnostics GmbH, Penzberg Germany; ^15^ UK Dementia Research Institute at UCL, London United Kingdom; ^16^ University of Wisconsin‐Madison, Madison, WI USA; ^17^ Roche Diagnostics International Ltd., Rotkreuz Switzerland; ^18^ School of Education, University of Wisconsin‐Madison, Madison, WI USA; ^19^ UCSF Weill Institute for Neurosciences, University of California, San Francisco, CA USA; ^20^ Wisconsin Alzheimer's Disease Research Center, University of Wisconsin School of Medicine and Public Health, Madison, WI USA

## Abstract

**Background:**

Less adequate cardiorespiratory fitness (CRF) is associated with several aspects of Alzheimer’s disease (AD) pathology, including neuroinflammation, neurodegeneration and synaptic dysfunction, all of which are known contributors to the clinical outcome – progressive cognitive decline [1]. AD‐associated biomolecular changes also seem to be attenuated in carriers of the functionally advantageous variant of the KLOTHO gene (KL‐VS_HET_) [2]. While KL‐VS_HET_ and CRF both appear to mitigate aspects of AD pathology, they have been exclusively studied in isolation. Here we investigate whether the relationships between CRF (VO_2_ max) and cerebrospinal fluid (CSF) biomarkers of neurodegeneration, synaptic dysfunction, and inflammation differ for KL‐VS_HET_ compared to non‐carriers (KL‐VS_NC_).

**Method:**

The cohort, enriched for AD risk, consisted of cognitively unimpaired adults (N=132; Mean_AGE_=62.7) from the Wisconsin Registry for Alzheimer’s Prevention and Wisconsin Alzheimer’s Disease Research Center. Covariate‐adjusted (age, sex, parental AD history, APOE, and age difference between CSF sampling and exercise test) linear models examined the relationship between VO_2_ max and CSF biomarkers of neurodegeneration [α‐synuclein (α‐syn), neurofilament light polypeptide (NfL), total tau (tTau)], synaptic dysfunction [neurogranin (Ng)], and neuroinflammation [glial fibrillary acidic protein (GFAP), soluble triggering receptor expressed in myeloid cells (sTREM2), chitinase‐3‐like protein 1 (YKL‐40), interleukin 6 (IL‐6), S100 calcium‐binding protein B (S100B)] as a function of KLOTHO KL‐VS.

**Result:**

The interaction between VO_2_ max and KL‐VS_HET_ was significant for tTau (P=0.05), Ng (P=0.02), sTREM2 (P=0.02), and YKL‐40 (P=0.03) (Table 1; Figure 1), such that KL‐VS_HET_ who were more fit had significantly lower levels of tTau, Ng, sTREM2, and YKL‐40 but not α‐syn, NfL, GFAP, IL‐6, or S100B (all Ps>0.63).

**Conclusion:**

We report a synergistic relationship between KL‐VS_HET_ and CRF with regard to neurodegeneration (tTau), synaptic dysfunction (Ng) and inflammation (sTREM2 and YKL‐40), suggesting a protective role for both KL‐VS_HET_ and better cardiovascular fitness against unfavorable AD‐related changes. Their potentially shared biological mechanisms will require future investigations.

**References**

[1] Huuha, A.M., et al., Can exercise training teach us how to treat Alzheimer's disease? Ageing Res Rev, 2022. 75: p. 101559.

[2] Driscoll, I., et al., AD‐associated CSF biomolecular changes are attenuated in KL‐VS heterozygotes. Alzheimers Dement (Amst), 2022. 14(1): p. e12383.